# Upper Brachial Artery Access Using a 4-French Sheath for Diagnostic Cerebral Angiography Prior to Transradial Neurointerventional Therapy: A Single-Center Retrospective Study

**DOI:** 10.7759/cureus.102232

**Published:** 2026-01-24

**Authors:** Taigen Sase, Hidemichi Ito, Kiyotaka Wakatsuki, Gaku Hidaka, Homare Nakamura, Hidetoshi Murata

**Affiliations:** 1 Department of Neurosurgery, St. Marianna University School of Medicine, Yokohama Seibu Hospital, Yokohama, JPN; 2 Department of Neurosurgery, St. Marianna University School of Medicine, Kawasaki, JPN

**Keywords:** 4-french sheath, access-site complication, diagnostic cerebral angiography, transbrachial access, transradial neurointervention

## Abstract

Purpose: The transradial access (TRA) is often preferred for neurointerventional therapy; however, radial artery occlusion after diagnostic angiography may limit subsequent TRA. To preserve radial access for neurointerventional therapy, this study proposed using the right transbrachial access (TBA) with a 4-French (Fr) sheath as a method for diagnostic angiography.

Materials and methods: A retrospective analysis was performed on 51 patients who underwent diagnostic cerebral angiography via the right TBA and subsequently received neurointerventional therapy using right-sided TRA between April 2023 and March 2025. In all cases, a 4-Fr short sheath was inserted into the right upper brachial artery for angiography of the ipsilateral forearm and brachial arteries. If anatomical anomalies were noted, additional left-arm angiography was conducted to assess the feasibility of left-sided TRA during future therapy.

Results: The mean age of the participants was 67.9 years, and 19 were women. Minor and transient events included puncture-site pain (3.9%) and transient forearm numbness (3.9%), and analgesics were used in 5.9% of cases. There was no subcutaneous hematoma or hemorrhagic event requiring intervention. On angiography during neurointerventional treatment, one (2.0%) patient presented with mild stenosis at the brachial puncture site, and no procedure-related issues were observed. No pseudoaneurysm, arteriovenous fistula, or hematoma with swelling was detected.

Conclusions: The use of the TBA strategy with a 4-Fr sheath in preoperative diagnostic angiography preserves radial artery patency while allowing anatomical evaluation. Hence, this method is a relatively safe and feasible option for centers adopting TRA-based neurointervention.

## Introduction

In recent years, the transradial access (TRA) has emerged as an interesting alternative to the traditional transfemoral approach for neurointerventional procedures. The TRA has several advantages, such as fewer access-site complications and earlier mobilization, which are beneficial in terms of both patient safety and comfort [1-3]. Its use is further supported by widespread adoption in cardiovascular interventions [4,5].

However, TRA-specific complications such as radial artery occlusion (RAO) remain a concern, and even diagnostic angiography via the TRA may cause RAO [6-9]. In cerebral angiography using the TRA, the incidence of RAO has been reported to be approximately 5.9%, and asymptomatic local complications, including access-site issues, have been reported in 9.6% of cases [7,8]. Although it is often asymptomatic, RAO may compromise future TRA access - an important concern for centers routinely using TRA. Hence, it is important to develop methods that can preserve the radial artery during diagnostic catheter examinations. In our institution, to achieve this goal, we perform diagnostic cerebral angiography via the transbrachial access (TBA) rather than the TRA, allowing preservation of the radial artery for subsequent neurointervention.

Herein, we report our institutional strategy-elective diagnostic cerebral angiography using the right TBA with a 4-Fr sheath, which reserves the radial artery for neurointervention. If anatomical variants (e.g., high-origin radial artery and aberrant right subclavian artery (ARSA)) were detected, additional left arm angiography was performed during the right TBA procedure to assess the left TRA as a possible access route for future neurointerventional procedures. The primary aim of this study was to describe our institutional preoperative diagnostic strategy using the right TBA and to evaluate its safety and feasibility based on access-site-related complication rates.

## Materials and methods

**Figure 1:**
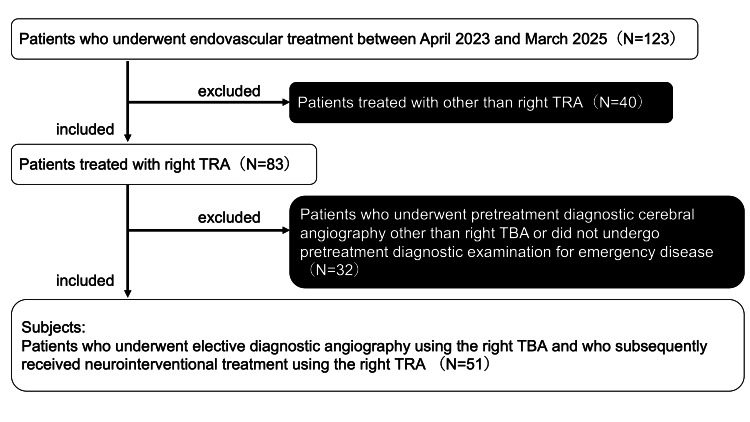
Study design and patient selection TBA: transbrachial access; TRA: transradial access

Study design and patient selection are summarized in Figure 1. This single-center, retrospective observational study included patients who underwent elective diagnostic angiography using the right TBA and who subsequently received neurointerventional treatment using the right TRA between April 2023 and March 2025. By applying these inclusion criteria, the punctured brachial artery during diagnostic angiography could be clearly evaluated angiographically at the time of neurointerventional treatment via the TRA. Emergency cases without preoperative diagnostic angiography were excluded from the analysis. During the study period, 123 neurointerventional procedures were performed at our institution. In total, 83 (67.5%) patients underwent the procedure using the TRA, and 51 patients met the inclusion criteria. Because diagnostic cerebral angiography was routinely performed via TBA during the study period, no comparable diagnostic TRA cohort was available for analysis.

Diagnostic angiography protocol

**Figure 2:**
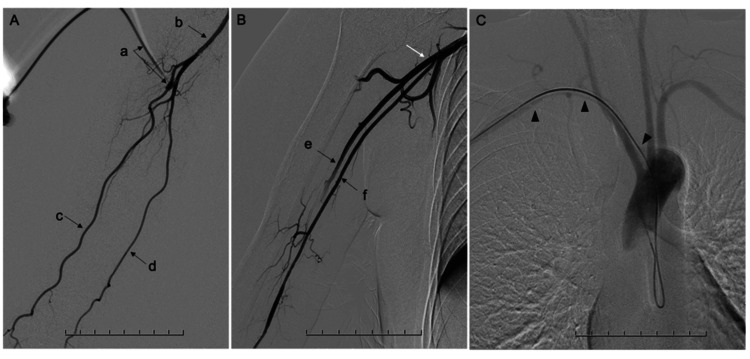
Transbrachial angiographic examples (A) Right brachial artery angiography using a 4-Fr sheath showing a normal radial-ulnar bifurcation (a: sheath, b: brachial artery, c: radial artery, and d: ulnar artery). (B) Example of the brachioradial artery (e: brachioradial artery, f: ulnar artery, and white arrow: high radial-ulnar bifurcation). (C) Example of the aberrant right subclavian artery (arrowheads).

Diagnostic angiography procedures are summarized in Table 1. All procedures were performed by neurointerventionalists certified by the Japanese Society for Neurovascular Therapy. Local anesthesia was achieved by subcutaneous injection of approximately 1 mL of lidocaine at the puncture site. Procedural sedation was not routinely administered during diagnostic angiography. As a contrast medium, iopamidol diluted to approximately 70% with normal saline was manually injected, with a total volume of approximately 5 mL at an injection rate of about 2 mL/s for brachial and arch angiography. Fluoroscopic imaging was performed using standard institutional settings. The TBA was obtained under palpation, and ultrasound guidance was used when necessary to minimize puncture attempts. A 4-French (Fr), 7-cm introducer sheath was inserted, followed by contrast injection through the sheath to evaluate the ipsilateral forearm arteries and the brachial artery, as shown in Figures 2A-2B. A 0.035-inch guidewire and a 4-Fr Simmons catheter were advanced, the Simmons curve was formed in the aortic arch, and arch angiography was performed to evaluate arch type and anomalies, including ARSA, as shown in Figure 2C. Selective angiography of head and neck vessels was then conducted according to the target pathology. During diagnostic catheterization, when anatomical variants or access difficulty were anticipated, additional left forearm and brachial angiography was performed to assess the feasibility of left TRA. Finally, the sheath was removed, and hemostasis was achieved using an air-inflated compression brachial band for patent hemostasis.

**Table 1:** Diagnostic angiography protocol This protocol represents an original, institution-developed approach developed by the authors based on institutional clinical experience. ARSA: aberrant right subclavian artery; TRA: transradial access

Seven-step transbrachial diagnostic angiography protocol	
1. Arterial access	In principle, right brachial artery puncture under palpation. If necessary, promptly switch to ultrasound-guided puncture.
2. Sheath insertion	Insert a 4-French, 7 cm introducer sheath.
3. Forearm and brachial artery angiography	Through the sheath, an angiography of the right forearm artery (radial, ulnar, interosseous arteries, etc.) is performed (Figure 2A). Next, an angiography of the right upper arm artery is performed to confirm the presence or absence of high branching of the radial artery and other anomalies (Figure 2B).
4. Catheter navigation	An aortic arch angiography is performed to evaluate the aortic arch type and confirm the presence of abnormal anatomies such as ARSA (Figure 2C).
5. Selective target vessel angiography	-
6. Left forearm and upper arm angiography (as needed)	The catheter is inserted into the left subclavian artery, and an additional arteriography of the left arm is performed to evaluate the possibility of left TRA in the future.
7. Sheath removal and hemostasis	Patent hemostasis is achieved with a compression brachial band with an air-release function.

Outcome measures

This study assessed the safety and validity of the right TBA for diagnostic angiography. Puncture-site-related events, including pain, the need for analgesic medication, forearm numbness, and hematoma formation, were assessed during hospitalization. Although no imaging follow-up was performed between the diagnostic angiography and the subsequent neurointerventional procedure, all patients underwent clinical evaluation of the puncture site at their first outpatient visit after discharge. In addition, vascular complications such as stenosis at the brachial puncture site, pseudoaneurysm, and arteriovenous fistula were evaluated angiographically on the day of the neurointerventional treatment. All data were collected through a detailed review of procedural logs, angiographic findings, and clinical records.

Ethical considerations

The current study was performed in accordance with the Declaration of Helsinki and was approved by the Ethics Committee of St. Marianna University School of Medicine, Yokohama Seibu Hospital (approval no. 6455). Written informed consent was obtained from all patients for the clinical procedure.

## Results

In total, 51 patients met the inclusion criteria (Table 2). The mean age of the participants was 67.9 years (range, 33-86 years), and 19 (37.3%) were women. The mean body mass index (BMI) was 23.0 kg/m^2^, and 27 patients (52.9%) were receiving antithrombotic therapy. The most common target diseases were cerebral aneurysms and cervical internal carotid artery stenosis. Regarding atherosclerotic risk factors, hypertension was present in 33 patients, dyslipidemia in 34, diabetes mellitus in 13, and hyperuricemia in seven.

**Table 2:** Baseline characteristics of the 51 patients

Patient's characteristics	Values
Age, mean (range), years	67.9 (33-86)
Sex	19 female (37.3%)
Body mass index, mean (range)	23.0 (15.5-31.8)
Antithrombotic medication, n (%)	27 (52.9)
Single antiplatelet therapy	17
Dual antiplatelet therapy	9
Direct oral anticoagulants	1
Atherosclerotic risk factors, n (%)	
Hypertension	33 (64.7)
Dyslipidemia	34 (66.7)
Diabetes mellitus	13 (25.5)
Hyperuricemia	7 (13.7)
Target disease for neurointervention, n (%)	
Cerebral aneurysm	19 (37.3)
Cervical internal carotid artery stenosis	24 (47.1)
Dural arteriovenous fistula/arteriovenous malformation	5 (9.8)
Meningioma	3 (5.9)

**Figure 3:**
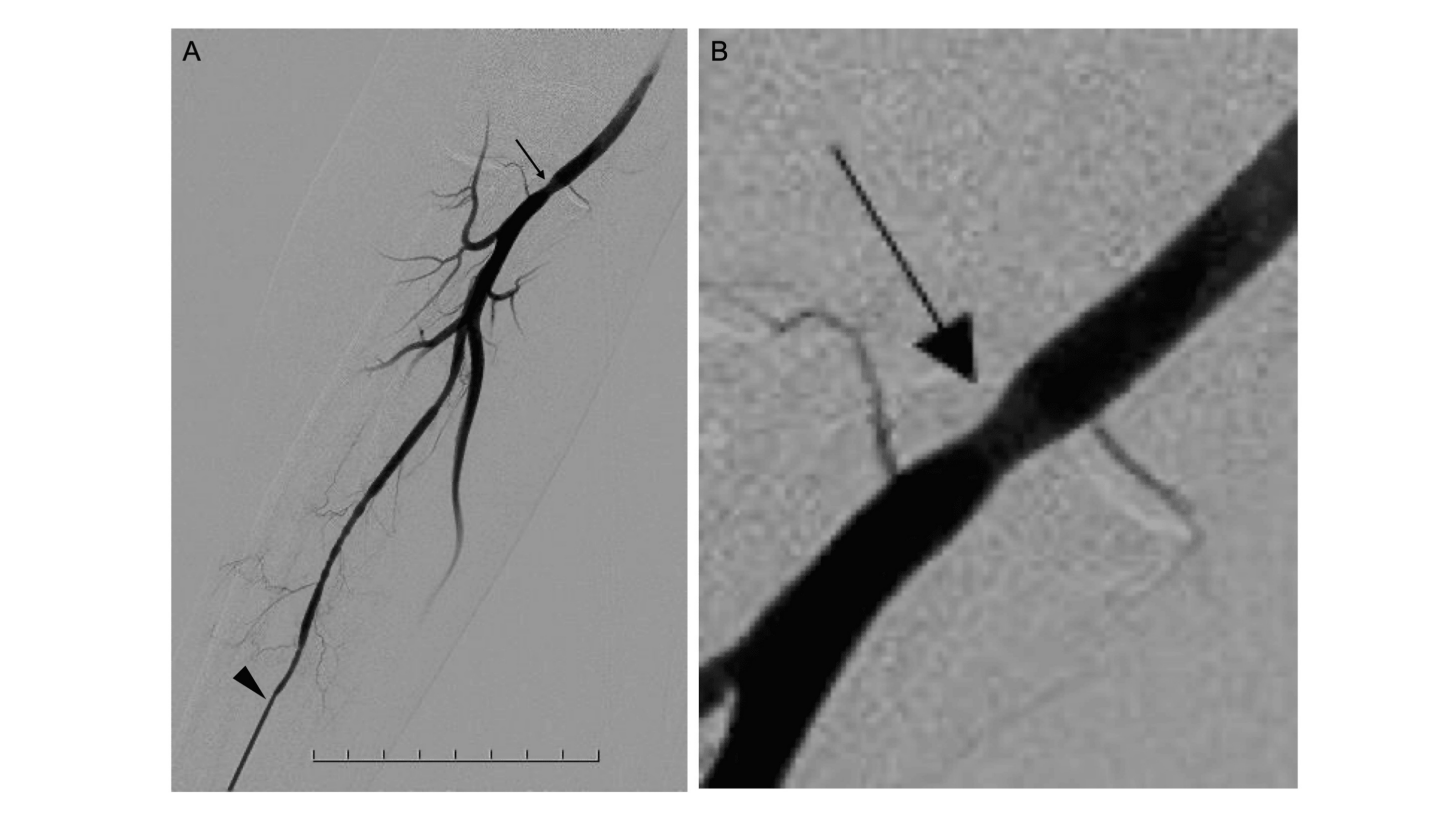
Mild brachial artery stenosis at the puncture site (A) One patient presented with mild brachial artery stenosis (arrow) at the puncture site of diagnostic catheterization on angiography during transradial neurointervention (arrowhead: sheath in the radial artery). (B) Enlarged image of the stenosis (arrow).

The principal outcomes are summarized in Table 3. All cases achieved successful puncture of the brachial artery. Ultrasound guidance was required in two (3.9%) cases, both of which involved multiple puncture attempts. Following diagnostic angiography via the right TBA, three patients (5.9%) required analgesic medication for mild puncture-site discomfort. One female patient (2.0%) with a BMI of 24.5 kg/m^2^ who was on dual antiplatelet therapy experienced delayed removal of the hemostasis band due to prolonged bleeding, but hemostasis was achieved without additional intervention. Two patients (3.9%) reported localized puncture-site pain, which resolved within one day. Transient numbness of the ipsilateral forearm was observed in two additional patients (3.9%), both of whom resolved spontaneously within one hour. One patient with dementia required physical restraint for behavioral reasons but exhibited no puncture-site complications. No palpable subcutaneous hematoma, swelling, or clinically significant bleeding was detected in any patient. At the first outpatient visit after discharge (mean, 21 days later), all patients underwent inspection and palpation of the puncture site, and no local abnormalities were identified. No pseudoaneurysm, arteriovenous fistula, or other vascular abnormalities were observed during follow-up or treatment. During subsequent TRA neurointervention, which was performed on a mean of 56 days after the diagnostic angiography, angiographic assessment of the brachial artery demonstrated mild stenosis at the previous puncture site in one patient (2.0%) (Figure 3), which did not interfere with the neurointerventional procedure. This angiographic finding is illustrated in Figure 3.

**Table 3:** Procedural outcomes and complications related to right transbrachial artery

Outcome/Complication	N (%)
During the catheter examination	
Successful procedure via right upper brachial artery puncture	51 (100)
Ultrasound-guided puncture required	2 (3.9)
After the catheter examination, on the next day	
Transient puncture-site-related events	4 (7.8)
Puncture-site pain	2 (3.9)
Transient forearm paresthesia	2 (3.9)
Permanent complications	0 (0.0)
Subcutaneous hematoma with swelling	0 (0.0)
Difficulty in hemostasis when releasing the pressure band	1 (2.0)
Local puncture-site abnormalities at first outpatient follow-up (mean, 21 days after diagnostic angiography)	0 (0.0)
Vascular complications following puncture of the right brachial artery (evaluation during transradial neurointervention; mean, 56 days after diagnostic angiography)	1 (2.0)
Mild stenosis at the punctured right brachial artery (Figure 4)	1 (2.0)
Pseudoaneurysm at the punctured right brachial artery	0 (0.0)
Arteriovenous fistula at the punctured right brachial artery	0 (0.0)
Others	0 (0.0)

Although not included in the present cohort, among the 123 neurointerventional procedures performed during the same period, eight were conducted via the left TRA. Of these, six underwent preoperative diagnostic TBA angiography using the protocol depicted in Figure 2 to evaluate the suitability of the left TRA. These cases demonstrated the usefulness of our diagnostic angiography protocol, which includes angiographic evaluation of the contralateral upper limb when necessary.

## Discussion

Based on our experience, the use of the right TBA with a 4-Fr sheath for preoperative angiography before TRA neurointervention is a relatively safe and feasible option. The complication rate was low, with only one case of mild stenosis at the diagnostic puncture site of the right brachial artery, and without significant adverse events.

Historically, TBA has been associated with higher complication rates (2-8%) compared with TRA or traditional transfemoral approach [10-14]. Nevertheless, our institutional protocol and operator expertise (performed by neurointerventionalists certified by the Japanese Society for Neurovascular Therapy) likely contributed to the reduced complication rates. The absence of pseudoaneurysms or other puncture-site complications at the brachial artery may be attributable not only to operator expertise but also to the fact that multiple punctures were strictly avoided. In addition, a previous study has shown that smaller-caliber sheaths further decrease complications [14]. The exclusive use of 4-Fr sheaths may have contributed to the favorable outcome.

One patient with delayed hemostasis was on dual antiplatelet therapy, and this finding indicated that antithrombotic therapy is a possible risk factor. The use of even smaller sheaths or the adjustment of the hemostasis protocol should be considered. In vascular interventions via the femoral access, smaller sheaths have reduced hemostasis time and length of bed rest [15].

Ultrasound-guided arterial access may also lower complication rates [16]. Even with guidance, TBA is associated with a non-trivial complication rate of approximately 2.7% for major events [13]. Ultrasound guidance was used selectively to reduce puncture attempts.

A major benefit of the right TBA for diagnosis is the preservation of radial artery patency. Local access-site complications, including RAO, with a rate of 1.8-9.5% in TRA angiography, can compromise later access [6-9]. Our approach aims to decrease that risk.

A comprehensive anatomical mapping during diagnostic angiography was also utilized to identify variants such as ARSA, high-origin radial artery, and tortuous subclavian/innominate arteries. In such cases, left-arm angiography during the same session allows for the evaluation of left TRA feasibility. We have previously reported the performance of safe left TRA embolization of a meningioma feeder via the ARSA [17].

The limitations of the current study include its single-center retrospective design and modest sample size. Moreover, because diagnostic cerebral angiography was routinely performed via the TBA during the study period, no comparable diagnostic TRA cohort was available for analysis. Therefore, the findings should be interpreted as preliminary and hypothesis-generating data, rather than as definitive evidence. Future studies with larger sample sizes are warranted to validate these findings. In addition, detailed anatomical variations and comprehensive vascular access-related complication profiles were not fully evaluated, which represents another limitation of the present study.

Based on our experience, diagnostic catheter examinations via the right TBA and treatment via the right TRA appear to be a relatively safe and reasonable option, which may theoretically contribute to radial artery preservation.

## Conclusions

This study evaluated the validity of a strategy using TBA diagnostic angiography prior to TRA neurointerventional therapy, which is the preferred approach at our institution. In the study cohort, the incidence of puncture-site complications was low, with no events resulting in permanent sequelae, and the radial artery was preserved. In addition, by assessing the right forearm and upper brachial arteries and aortic arch during the diagnostic phase and incorporating left forearm and upper brachial angiography procedures as necessary, the selective use of the left TRA became feasible. Diagnostic angiography using the right TBA with a 4-Fr sheath appears to be a relatively safe and rational method for preserving radial access and allowing a comprehensive anatomical evaluation. Our findings suggest that this method may help reduce the risk of RAO and could contribute to more flexible and safer procedural planning in neurointervention.
